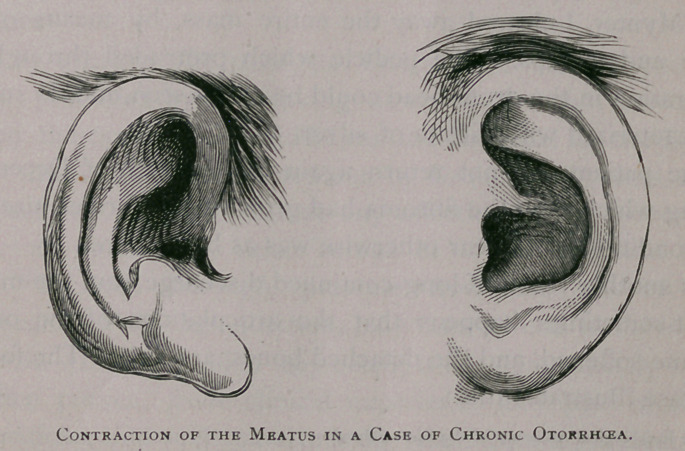# Chronic Purulent Inflammation of the Middle Ear

**Published:** 1879-12

**Authors:** Lucien Howe


### THE EFFECTS OF CHRONIC PURULENT INFLAMMATION OF THE MIDDLE EAR.

BY LUCIEN HOWE, M. D.

In the August number of this Journal the writer reported some results of the treatment of chronic purulent inflammation of the middle ear, by means of the permanganate of potassa; and the favorable reception given to that article is the only excuse for so soon presenting another on a similar topic.

Since, if it is well to record observations indicating a reliable method for controlling this disease, it can hardly be of less interest to call attention to its results, when left uncared for. These are apparently so obvious and often considered of so little importance that they may seem hardly worthy of serious consideration.

The popular notion is unfortunately too prevalent that a discharge from the ear will surely cease, and that temporary deafness or a disagreeable odor is the only disadvantage accompanying it. Moreover, even physicians are occasionally heard to intimate that it should not be "meddled with," for if suddenly arrested, it may "strike in upon the brain."

The fallacy of thus mistaking cause for effect will be referred to presently, but such remarks only show that no opportunity should be lost to state the truth, and fortify it by examples. For practitioners who know this disease best, are those who fear it most. If there is any one topic concerning which authorities are thoroughly agreed, it is in regard to the evil effects of a long-neglected purulent discharge from the middle ear.*

In regard to this disease Sir William Wilde says: "We can never tell how, when, or where it will end." The chapter concerning it is one of the best in his excellent work, and together with other writers, he has presented the subject so clearly and so forcibly as to leave but little for any one else to say. It would be useless, therefore, to go into the detailed consideration of pathology and treatment, which can easily be found, in any text-book on the ear. But it seems that the effects of this disease might be made prominent by simply describing a few cases. Examples similar to those which will be referred to have imbued writers with the strong convictions they often express, and perhaps others will make the same deductions when the data are thus enumerated.

It will be understood, of course, that the accompanying cases are illustrative of the results which occur not ordinarily, but only in exceptional instances—some of them very rarely. A few of these have already been referred to in the article on permanganate of potassa, as being incurable by that method, or as becoming discouraged before any conclusions could be drawn as to its effect. The others were treated differently, or were seen before any systematic trials were made with that remedy.

Before specifying these cases, however, it may be well to recall for a moment the anatomical relations of the part under consideration. It will be remembered, of course, that the cavity called the middle ear is situated on the inner side of the drum-head that membrane forming its outer wall. In the normal condition this is a structure of extreme tenuity, and when dissected out resembles a piece of tissue paper, but when thickened by inflammatory processes, may become quite firm and resisting.

* St. John Roosa, Diseases of the Ear, page 369.

Opposite the drum-head, we find on the inner wall of the cavity a thin layer of bone, which separates the labyrinth from the middle ear, and also two openings into the internal ear, one of which, in fresh preparations, is closed by the secondary membrana tympani, and the other by the oval plate of the stapes. This inner wall at one portion separates the middle ear from the facial nerve, and at another, where, moreover, it is often perforated by innumerable openings for vessels and nerves, it constitutes the main barrier between that cavity and the carotid artery. On the anterior wall we find principally the orifice of the Eustachian tube, and posteriorly the mastoid cells, which also open into this cavity.

The lower wall or floor is usually formed by a thin plate of bone, but not infrequently it is partly or wholly membranous, while just beneath it lies the jugular vein. The roof is still more important, separating as does the middle ear from the brain.

When describing this portion, Dr. Burnett says: "This osseous partition is very thin, and in some cases congenital fissures in it persist; in such instances the only boundary at the dehiscences, between the tympanum and the cerebral cavity, is formed by the mucous membrane of the former, and the membranes of the brain. It is evident that in such cases pathological processes in the drum cavity are especially liable to pass upward to the brain." *

The cavity of the middle ear, with the chain of delicate bones contained in it, is, therefore, placed in the closest proximity to portions of vital importance, and we might infer a priori that a suppurative inflammation of its lining membrane would not only prove detrimental to hearing, but injurious to the contiguous structures.

For by the term chronic purulent inflammation of the middle ear, we understand such a morbid condition of the mucous membrane lining that cavity as can be found also in other portions of the body. In an early stage it is injected, swollen and

* A Treatise on the Ear, Burnett, page 79.

covered with a glairy fluid, and then, as the catarrhal stage becomes more advanced, accompanied by symptoms more or less acute, the secretion may assume a distinctly purulent character.

For awhile the pus remains shut up within the walls of the tympanum, except as a portion oozes out through the Eustachian tube. But ordinarily, the walls of that passage become also swollen by the irritating secretion which flows through it, until this single door of escape becomes closed, and then with symptoms of varying intensity the accumulating fluid pushes in the direction of least resistance. The drum-head is usually the first one of the six walls to give way, a foetid discharge is poured into the outer canal, and a purulent inflammation is fairly established, which may afterwards become "chronic."

And this process is invariably a destructive one. The longer it lasts the greater the detriment to the ear, and the less the prospects for improvement. In considering the evils which follow this disease, it is perhaps well to mention first the impairment of hearing. This varies as to degree and duration, according to the extent which the process has reached. In some instances it is principally due to the vibrations of the drum-head being impeded by an accumulation of secretion.

The use of a syringe will at once improve such cases. But more frequently the difficulty in hearing is dependent upon the extensive destruction of the membrane, or upon a diseased condition of the chain of bones. A perforation of the drum-head does not, however, necessarily produce deafness. The error of this notion was proven by Sir Astley Cooper long ago.* Cases continually occur in which only a portion of the drum-head remains and the person is still able to hear quite well.

On the other hand, where the perforation is very small the deafness is sometimes extreme, being due to an impeded action of the chain of bones, or to other results of the inflammation.

The following case illustrates this condition: A girl, 14 years old, was brought to me on the 12th of October, from Le Couteulx St. Mary's Institution for the instruction of deaf mutes.

---

* Philosophical Transactions of the Royal Society of London, 1801.

When a child, nothing unusual was observed in regard to her hearing. About 10 years ago an inflammatory disease of the ears began, which assumed the purulent form, became chronic, and finally left her entirely deaf. An examination showed the drum-head on each side to be considerably swollen and perforated, but in neither ear was the opening much more than a millimeter in diameter. She had, however, become a deaf mute, and even the vibrations of the tuning fork, when placed on the forehead, could with difficulty be perceived as a sound. Moreover, it appeared upon inquiry that this was not the only inmate of the same institution whose unfortunate condition could be traced to a similar cause. The Sister of Charity in charge very kindly furnished me with such information as could be gathered from the records and otherwise, and it appeared that out of 132 pupils, nineteen had suffered from the disease under consideration, and that in twelve of these cases the discharge still persisted. Together with the impairment of hearing, the patient is always troubled by the disagreeable odor of the secreted pus. Those who have one good ear remaining are often more annoyed by the discharge than by the deafness. In most instances it is simply troublesome, and can be, to a great extent, obviated by frequent washings and by filling the ear with a plug of cotton; but occasionally the stench becomes intolerable.

In June, '78, a boy applied for relief at the Buffalo Eye and Ear Infirmary, who had been twice sent away from school on this account. Although the disease affected only one ear, the atmosphere in his immediate vicinity was positively sickening, and even at a distance of several feet from the child the perfume was painfully apparent.

Besides the disagreeable odor of the discharge, it is frequently of an irritating character. An eczematous eruption may appear about the auricle, or ulcerations be produced, with their consequent cicatrices and deformities.

Such a result was observed in a patient who applied at the Infirmary on the 23d of last August. A boy, Robert R., eight years old, complained of a discharge from the right ear, which had lasted for about three years. It appeared, from the account given by the mother of the patient, that the exuded pus varied somewhat both as to quantity and quality, and at times seemed to be particularly irritating.

The resulting condition, as regards the auricle, was rather unique, for when the two were compared they were as unlike as though belonging to different individuals. That on the left side was rather large, well shaped, and with a wide opening for the meatus. On the right side, however, the helix and anti-helix were drawn forward, the concha narrowed, the anti-tragus raised almost perpendicularly, giving the lobule an elongated appearance, while the opening for the meatus was contracted into an oval fleshy ring, which measured only five millimeters in the horizontal diameter and four millimeters vertically. A mucous polypus, moreover, occupied most of the canal. Altogether it seemed that such a case was worthy of being recorded rather more exactly than by simple description, and accordingly, a photograph was taken of each auricle, from which the accompanying wood cuts were copied. And just as the pus is injurious to external parts, so does it affect the structures, as already intimated, which are situated within, or contiguous to, the cavity of the middle ear.

Contraction of the Meatus in a Case of Chronic Otorrhœa.

The mucous membrane being the part primarily involved, naturally suffers most. Not only does it become engorged and swollen, but projecting outward, a portion may push through the opening in the drum-head and thus form a polypus. In the great majority of cases there are soft masses of tissue (mucous polypus) "being produced," as Swartz says, "by a hyperplasia of the tympanic mucous membrane."* Occasionally, however, as the chronic purulent discharge continues, the periosteal layer of the middle ear develops abnormally in a similar manner, and pushing outwards, protrudes externally as a dense yellowish tumor—a real fibroma.

I have found these much more difficult of removal than the more common mucous polypi, and apparently with a marked tendency to recur.

A typical case of this sort presented itself at the Eye and Ear Infirmary, on the 31st of May, 1876. The patient, J. L., was otherwise a healthy young fellow, complaining only of the deafness and offensive discharge, for about two years. For some weeks past he had noticed "that lump" growing out from his ear, and an examination showed it to be a fibroma. An attempt was made to remove it in the usual way with Wilde's snare. This, however, proved so difficult and painful that on June 4th the patient was chloroformed, and assisted by Dr. Herman Mynter, I cleared away the entire mass, by means of the snare and forceps. The pedicle which protruded through the perforation in the drum-head could be easily seen, and its surface was cauterized with nitrate of silver.

The patient did not return again till the 27th of December, during which time the fibroma had grown to its former size, and the condition of the ear otherwise was as bad as ever.

As another result of long-continued discharge from the middle ear, it sometimes happens that the articulations of the osicles become softened, and the detached bones are lost. The following case illustrates this:

* The Pathological Anatomy of the Ear—Schwartz.

In July of 1875, I was consulted in regard to G. W., the six-year-old son of a clergyman in this city. The father stated that on the first of January of that year the patient showed symptoms of scarlet fever, which in due time developed into a severe type of that disease. On the 12th day both ears began to discharge, and continued from that time to secrete an acrid pus, frequently mixed with blood. About the first of April one of the bones of the right ear was accidentally found on the patient's pillow. Another subsequently lodged in the external canal, and finally the family physician, Dr. S. W. Wetmore, obtained the entire set, as beautifully cleaned by long maceration as though they had been boiled in a solution of caustic potash. Meanwhile the other ear had become affected to a similar degree, and the child was reduced practically to the condition of a deaf mute.

The resultsof the disease thus far considered, with the exception of the loss of function, manifest themselves externally. When, however, the accumulating pus cannot find exit along the usual routes, then the consequences may be still more serious. By its effect upon the Aqueductus Fallopii, a paralysis of the facial nerves may be produced; by infiltrating the mastoid cells, a destruction of that portion of the temporal bone may result; and finally, the death of the patient has not infrequently been observed by the transmission of the disease to the adjoining brain substance.

Let us consider the paralysis first. This is, of course, usually produced by a breaking down of the thin partition between the cavity of the middle ear and the canal for the facial nerve. But it has been pointed out by Gruber and others, that this result occurs also without any affection of the bone, by simply an infiltration, either serous or otherwise, into that canal, "or even through pressure from the side of the middle ear."* Although questions may thus arise as to the exact manner in which the lesion may occur, it has unfortunately been too often proven, from clinical experience and post mortem examinations, that such paralyses do result from a purulent in-

* Gruber—Lehrbuch der Ohrenheilkunde, page 540.

flammation of the middle ear. The following case shows how rapidly and completely this effect may be produced:

A young man, J. D., 19 years old, living at 483 Hickory street, applied to me Feb. 27, '78. His history, as written by himself and repeated by his father, was substantially as follows:

About six months previously, when traveling with a circus troupe, he began to suffer from a severe attack of "ear ache" on the right side. This, together with symptoms of malarial character and "fever," was sufficient to confine him to bed for a few days, during which time a similar process began in the other ear. As the pain subsided, an acrid and profuse discharge ensued, which continued in the left for about two months, and still persisted in the right.

Meanwhile the hearing was lost almost entirely, ordinary conversation could not be perceived at all, and the loudest tone was distinguished only as an indefinite sound. But a complication of hardly less importance was the paralysis of the right facial nerve. The eyelid was displaced, the corner of the mouth partly open, and the entire side of the face presented that vacant and stupid expression characteristic to this affection.

This, of course, is an unusual example. The canal, containing the nerve, is usually so thoroughly separated from the middle ear as to make such results quite rare. An inflammation and partial destruction of the mastoid cells is, however, more common. It will be remembered that they communicate directly with the cavity just mentioned, and a morbid process in one could be transmitted to the other, as, for example, in the following case:

J. L., a bright little girl five years old, had suffered since the middle of last May with a discharge from both ears—profuse and constant in the right, scanty and intermitting in the left. About the middle of September a mucous polypus developed in the right side, almost occluding the external canal, and later, an abscess formed behind the same ear and was allowed to open of its own accord. A repetition of the same process began soon after, and on the 29th of October, when the child was brought to me by the family physician, Dr. Loomis, the entire vicinity of the mastoid was swollen, tender and fluctuating. I therefore opened the abscess, allowing considerable pus to escape, and as the point of the knife struck the hard surface below, it passed along, giving that roughened, or gritty feel, peculiar to dead bone.

Finally, a chronic purulent inflammation of the middle ear has more than once taken on an acute form, and resulted in the death of the patient. Thus far, I have fortunately had no opportunity of seeing a case of this kind, but the testimony of others would tend to show that such examples occur to most practitioners of considerable experience. Nor is it at all surprising, when we remember how intimately the middle ear is related to structures of vital importance, especially the brain.

Concerning this, Turnbull* says: "The tissues adjoining the mastoid process, viz., the dura mater, the internal carotid artery, and internal jugular vein with the transverse and lateral sinus, are so important, that a morbid process which spreads to them from the middle ear is fraught with danger to life, independently of the circumstances that the sense of hearing may be weakened or entirely annihilated."

Burnett† says: "It becomes, therefore, the duty of every conscientious practitioner of medicine to be carefully observant of the onset of an inflammation in the mastoid cavity, and prompt to relieve it; for by so acting, he will, in all probability, save life where in similar cases there is every reason to know that death has occurred."

Dr. Albert H. Buck, one of the editors of the *American Journal of Otology*, considers the existence of such a disease, in certain instances, a sufficient cause for rejection of an applicant for life insurance, because, "these individuals, besides having to run the same chances of death, from diseases and accident, which other men must run, are afflicted with a local inflammation in very

*Lawrence Turnbull, Diseases of the Ear, page 195.

†Loc. Cit. page 545.

close proximity to the brain, an inflammation which may at any time excite a fatal meningitis, or by involving the veins of the neighborhood, lead ultimately to an embolism of some important arterial trig, or to the equally serious condition of septicæmia or pyæmia."*

It is only necessary to examine casually the literature relating to otology to assure one's self, from clinical histories and from post-mortem appearances, that fatal results of the disease under consideration are by no means so very rare. It would be out of place here to enter into bibliographical detail, but a few references to such cases may tend to show why they are to be regarded with suspicion.

The fact has long been recognized that a purulent inflammation of the middle ear may endanger life, but especial attention was directed to the subject by Von Troltsch,† whose unusual opportunities for extensive observation enabled him to collect fifteen cases of fatal otorrhoea, the dissections of which were carefully described.

About the same time‡ Dr. E. H. Bennet showed to the Pathological Society, of Dublin, two specimens illustrative of the fatal results of caries of the temporal bone. In Turnbull's "Diseases of the Ear" he reports the cases of a child and a young girl who died from the same cause; the diagnosis in both instances being confirmed by post-mortem examinations, and in Dr. Charles Burnett's work, about four pages are entirely devoted to the enumeration of articles "pertaining to the fatal results of neglected otorrhoea, caries of the mastoid, and the parts of the temporal bone, and operations on the mastoid portion."

Taking into consideration, therefore, such a series of observations, it would indeed appear that purulent inflammation of the middle ear is not, after all, such a simple affair as many are apt to regard it. The impairment of hearing, and disagreeable character of the discharge, are, at least, annoying to the patient,

*Medical Record, volume X, page 287.

† Archiv fur Ohrenheilkunde—Bd. IV, Heft II.

‡ British Medical Journal, Dec. 24, 1870. Medical News, Philadelphia, Feb., 1871.

and occasionally render him unfit for ordinary vocations. The resulting polypi, the paralyses, or inflammations of the bone, are of even more importance, and finally statistics indicate that a certain proportion of neglected cases terminate in the death of the patient. Such facts as these seem to be sufficient reason for thus reiterating and illustrating the results which sometimes follow a neglected otorrhoea.

## Figures and Tables

**Figure f1:**